# Electroacupuncture inhibits the expression of HMGB1/RAGE and alleviates injury to the primary motor cortex in rats with cerebral ischemia

**DOI:** 10.1515/tnsci-2022-0316

**Published:** 2023-10-09

**Authors:** Zeyin Nie, Huachun Miao, Chenyu Li, Feng Wu

**Affiliations:** Department of Human Anatomy, Wannan Medical College, No. 22, Wenchang West Road, Wuhu, 241002, Anhui, China

**Keywords:** cerebral ischemia, electroacupuncture, primary motor cortex, HMGB1, RAGE

## Abstract

**Background:**

The high-mobility group box 1 (HMGB1)/receptor for advanced glycation end products (RAGE) signaling pathway holds promise as a potential therapeutic target for ischemic brain injury. The effects of FPS-ZM1 and electroacupuncture (EA) on activation of the HMGB1/RAGE signaling pathway after cerebral ischemia remain uncertain.

**Methods:**

Middle cerebral artery occlusion (MCAO) model was established. Neurological function was assessed using Longa scores. Nissl staining was used to observe the morphology of neurons. The expression levels of HMGB1 and RAGE were assayed with immunofluorescence staining and western blot.

**Results:**

The results showed that EA and FPS-ZM1 could reduce the neural function score and neurons cell injury in cerebral ischemia rats by inhibiting the expression of HMGB1 and RAGE in primary motor cortex (M1) region. In addition, EA combined with FPS-ZM1 had a better therapeutic effect.

**Conclusions:**

The HMGB1/RAGE pathway could be activated after cerebral ischemia. Both EA and FPS-ZM1 improved neurological deficits and attenuated neuronal damage in rats. They had synergistic effects. These interventions were observed to mitigate brain damage by suppressing the activation of HMGB1/RAGE.

## 1 Introduction

Cerebral ischemia, also known as ischemic stroke, is a prevalent central nervous system disorder that leads to significant morbidity and mortality, accompanied by persistent neurological deficits [1]. The pathogenesis of cerebral ischemia is complex, and current clinical interventions are not fully satisfactory. In recent years, the high-mobility group box 1 (HMGB1)/receptor for advanced glycation end products (RAGE) signaling pathway has emerged as a promising target for therapeutic interventions in cerebral ischemia [2].

HMGB1 is a nuclear DNA-binding protein released passively by dying cells and can amplify the inflammatory cascade [3]. RAGE is a type 1 transmembrane glycoprotein [4]. While RAGE is widely distributed, it is primarily expressed in blood vessels and nervous tissues, where it can contribute to vascular injury by promoting inflammation and inducing endothelial cell apoptosis [5]. FPS-ZM1, a highly potent RAGE inhibitor, can penetrate the blood–brain barrier and has shown therapeutic potential in Alzheimer’s disease and Parkinson’s disease [6–8]. By reducing neuroinflammation, cell death, and neurological deficits through the ligand/RAGE/DIAPH1 pathway, FPS-ZM1 provides neuroprotection in rats with middle cerebral artery occlusion (MCAO) [9]. This potent antagonist of RAGE demonstrates considerable promise for the treatment of various inflammatory conditions, although clinical trials of this compound have not yet been reported [10].

Electroacupuncture (EA) is a safe and minimally invasive therapeutic technique that combines traditional Chinese acupuncture with electrical stimulation [11]. Numerous studies have demonstrated the potential of EA in mitigating brain injury caused by oxidative stress, improving poststroke neurological function, promoting autophagic clearance, and preventing ischemia-related damage in various organs [12,13]. Acupuncture at Baihui (GV20) and Zusanli (ST36) acupoints has been shown to enhance behavioral recovery in rats with cerebral ischemia/reperfusion injury, and these acupoints are commonly utilized in clinical practice for the treatment of ischemic stroke [14,15]. However, the precise mechanism through which EA exerts its neuroprotective effects in cerebral ischemia, particularly its modulation of the HMGB1/RAGE signaling pathway, remains unclear.

The primary motor cortex (M1) is situated anterior to the central sulcus [16] and serves as a crucial area for evaluating motor function in stroke patients [17]. In this study, we employed rats with MCAO to investigate the effects of EA, sEA, and FPS-ZM1 on the expression of the HMGB1/RAGE signaling pathway in the M1 region surrounding the ischemic focus.

## 2 Materials and methods

### 2.1 Animals

Ninety-six healthy male Sprague Dawley rats (8 weeks old, 230 ± 20 g) were obtained from Nanjing Qinglongshan Laboratory Animal Co., Ltd (Jiangsu, China). The animals were housed in a controlled environment with a temperature range of 22–24°C, a 12 h light/dark cycle, and *ad libitum* access to food and water.

### 2.2 Preparation of the cerebral ischemia model

After a 1-week adaptation period, the rats fasted for 12 h before surgery while still having access to drink. Anesthesia was induced by intraperitoneal injection of sodium pentobarbital at a dose of 30 mg/kg. The rats were positioned in a supine position on the operating table, and incisions were made in their necks to expose the right common carotid artery (CCA), external carotid artery (ECA), and internal carotid artery (ICA). A small incision was then made in the ECA to allow for the introduction of the embolization line (Cinontech, Beijing, China) into the ICA through the ECA. The embolization line was carefully secured in place, and the incision was closed with sutures. In the sham-operated (Sham) group, only the CCA, ECA, and ICA were exposed and isolated, without the insertion of an embolization wire.

### 2.3 Experimental protocol

The rats were divided into eight groups (*n* = 12) through random assignment: normal (Normal), Sham, MCAO, EA, sham EA (sEA), FPS-ZM1 (FPS), EA + FPS-ZM1 (EA + FPS), and sham EA + FPS-ZM1 (sEA + FPS) groups. Except for the Normal and Sham groups, all groups underwent MCAO surgery to induce cerebral ischemia. Five minutes after the surgery, the FPS group, EA + FPS group, and sEA + FPS group received intraperitoneal administration of 1 mg/kg of FPS-ZM1 (HY-19370, MedChemExpress, USA) once daily for 14 days. The dosage of FPS-ZM1 was determined based on previous research [9]. The EA group and EA + FPS group received EA stimulation at GV20 (2 mm oblique backward stab in the middle of the parietal bone) and left ST36 (7 mm straight stab approximately 5 mm below the small head of the fibula) 24 h after MCAO, with a low frequency of 2 Hz, a current of 1 mA, and a pulse width of 1 ms, for 30 min daily, following established references [14,18]. The sEA and sEA + FPS groups underwent subcutaneous piercing at GV20 and ST36, but the acupuncture needles were not electrically connected to the EA apparatus [19].

### 2.4 Neurological deficit assessment

Neurological function assessments were conducted at predetermined time intervals of 2 h, 1, 3, 7, and 14 days after the surgical procedures using the modified Longa scoring method. The scoring system consisted of the following criteria: 0 points denoting the absence of neurological deficit, 1 point indicating the incomplete extension of the contralateral forepaw, 2 points reflecting contralateral turning during ambulation, 3 points indicating contralateral leaning while walking, and 4 points representing an inability to spontaneously ambulate with varying degrees of impaired consciousness. Two physicians blinded to the treatment conditions independently evaluated the neurological scores of the subjects, with no knowledge of the experimental objectives.

### 2.5 Nissl staining

Neuronal density in the M1 region of the rat cortex was assessed using Nissl staining in each group (*n* = 6). Rats were intraperitoneally anesthetized with sodium pentobarbital (45 mg/kg), and their brains were fixed with 4% paraformaldehyde. Paraffin embedding was performed, followed by sectioning of 5 µm-thick coronal brain slices using a microtome. These sections were then subjected to sequential dewaxing with xylene and gradient alcohol. Nissl staining was performed using a commercial kit (DK0022, Leagene, China) that involved cresyl violet staining, immersion staining in a 56°C thermostat for 1 h, rinsing with double distilled water, and Nissl fractionation for 30 s. Subsequently, the slices were sealed with neutral balsam, and the number of neuronal cells was quantitatively analyzed using ImageJ software. Neuronal density was determined by counting the number of neurons in the M1 region of the rat cortex.

### 2.6 Immunofluorescence staining

**Figure 1:**
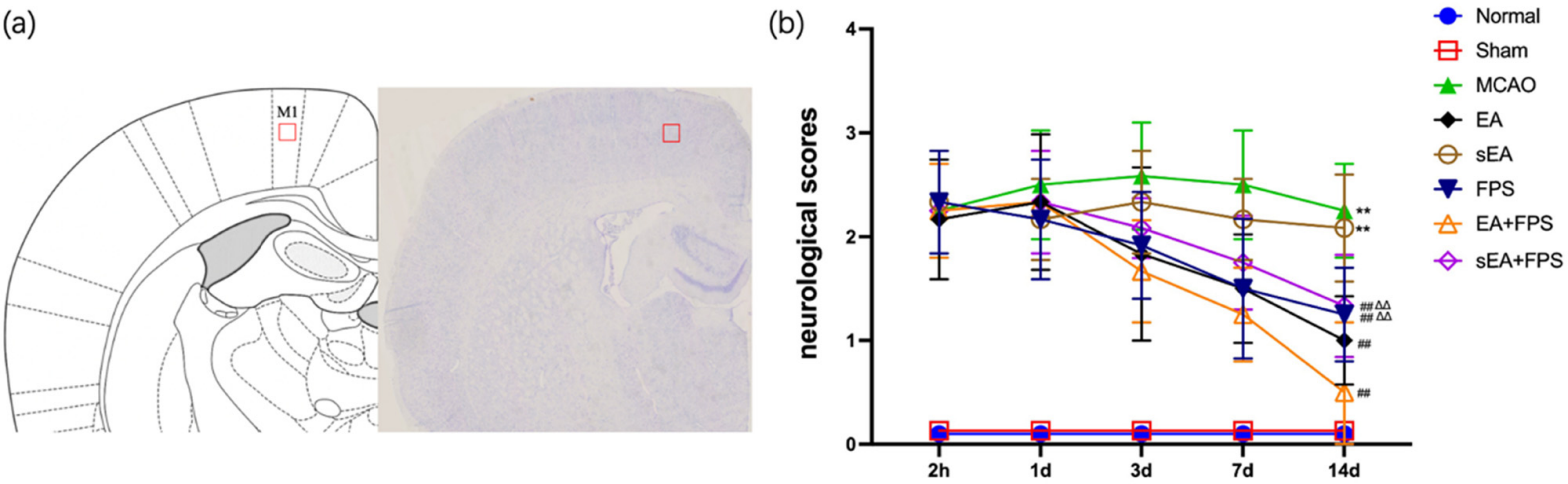
Schematic diagram and neurological scores. (a) Schematic diagram of the primary motor cortex (M1). (b) Neurological function scores of the eight groups at 2 h, 1 day, 3 days, 7 days, and 14 days after surgery using the Longa scale. *n* = 12/group. **p < 0.0001, MCAO vs Normal; **p < 0.0001, sEA vs Normal; ^##^ *p* < 0.0001, EA vs MCAO; ^##^ *p* < 0.0001, FPS vs MCAO; ^##^ *p* < 0.0001, EA + FPS vs MCAO; ^##^ *p* < 0.0001, sEA + FPS vs MCAO; ^∆∆^ *p* = 0.0018, FPS vs EA + FPS; ^∆∆^ *p* = 0.0003, sEA + FPS vs EA + FPS. ANOVA; mean ± SEM.

The sections were dewaxed and subjected to a citrate buffer solution for antigen repair. After reaching room temperature, H_2_O_2_ was added, and the sections were washed three times with PBS. Triton was then added, and the slices were incubated for 2 h without washing. Subsequently, the slices were incubated with HMGB1 antibody (1:200, 79823; Abcam, UK) or RAGE antibody (1:100, 216329; Abcam, UK). The following day, a fluorescent secondary antibody was added in the dark, followed by rinsing with PBS and the addition of DAPI. Finally, the slices were sealed. Evaluators blinded to the experiment used ImageJ to quantify the number of immunoreactive positive cells within a predetermined region (Figure 1a).

### 2.7 Western blot

The M1 region of the right cortex was collected from the ice box 14 days after treatment in each group (*n* = 6). Total tissue protein was then extracted and subjected to electrophoresis using 12% SDS-PAGE (Bio-Rad, Hercules, USA) with equal protein loading. The proteins were transferred onto PVDF membranes (FFP32, Beyotime, China) and blocked for 2 h in a blocking solution. The primary antibodies used included rabbit anti-HMGB1 (1:1,000, 79823; Abcam, UK), rabbit anti-RAGE (1:1,000, 216329; Abcam, UK), and rabbit anti-GAPDH (1:1,000, AF7021, Affinity, USA). The membranes were incubated with secondary antibodies (1:1,000, A0208, Beyotime, China) and subsequently scanned using a Gel-Pro system (Tanon Technologies, China). ImageJ software was utilized to measure the grayscale value of each band, and the relative expression of the target protein was determined by calculating the ratio of the target band to the internal reference band.

### 2.8 Statistical analysis

Statistical analysis was conducted using SPSS 18.0 for data analysis, and GraphPad Prism 9.0 was used for image plotting. Differences among three or more groups were assessed using ANOVA. The data were presented as mean ± standard deviation, and statistical significance was defined as *p <* 0.05.


**Ethical approval:** The research related to animals’ use has been complied with all the relevant national regulations and institutional policies for the care and use of animals. This study was conducted in compliance with the guidelines and regulations set forth by the Experimental Animal Welfare and Ethics Committee of Wannan Medical College, China (Approval Number: LLSC-2021-025). Adequate measures were implemented to ensure the welfare and minimize any potential discomfort experienced by the rats during the experimental procedures.

## 3 Results

### 3.1 Neurological deficit assessment

To assess the impact of acupoint EA, sEA, and FPS-ZM1 on the neurological recovery of MCAO rats, we employed the Longa scoring system to evaluate their behavior throughout the treatment period. The scores in the EA, FPS, EA + FPS, and sEA + FPS groups exhibited a gradual decline from Day 3 to Day 14, with notable differences observed among the groups on the 14th day. Notably, the Normal and Sham groups displayed no neurological impairment, while the MCAO and sEA groups exhibited the highest Longa scores, indicating significant differences compared to the Normal group (*p* < 0.01). These findings confirm the successful establishment of the MCAO model and demonstrate that sEA had no effect on the neurological function of rats with cerebral ischemia. Furthermore, the EA, FPS, EA + FPS, and sEA + FPS groups exhibited significantly lower neurologic function scores than the MCAO group (*p* < 0.01), indicating that EA and FPS-ZM1 effectively mitigated brain damage in MCAO rats after treatment. No significant difference was observed between the FPS and sEA + FPS groups, while the EA + FPS group exhibited significantly lower scores than the FPS and sEA + FPS groups (*p* < 0.01). This highlights the enhanced efficacy of combining EA with FPS-ZM1 compared to individual treatment with EA or FPS-ZM1 alone (Figure 1b).

### 3.2 Results of Nissl staining

**Figure 2:**
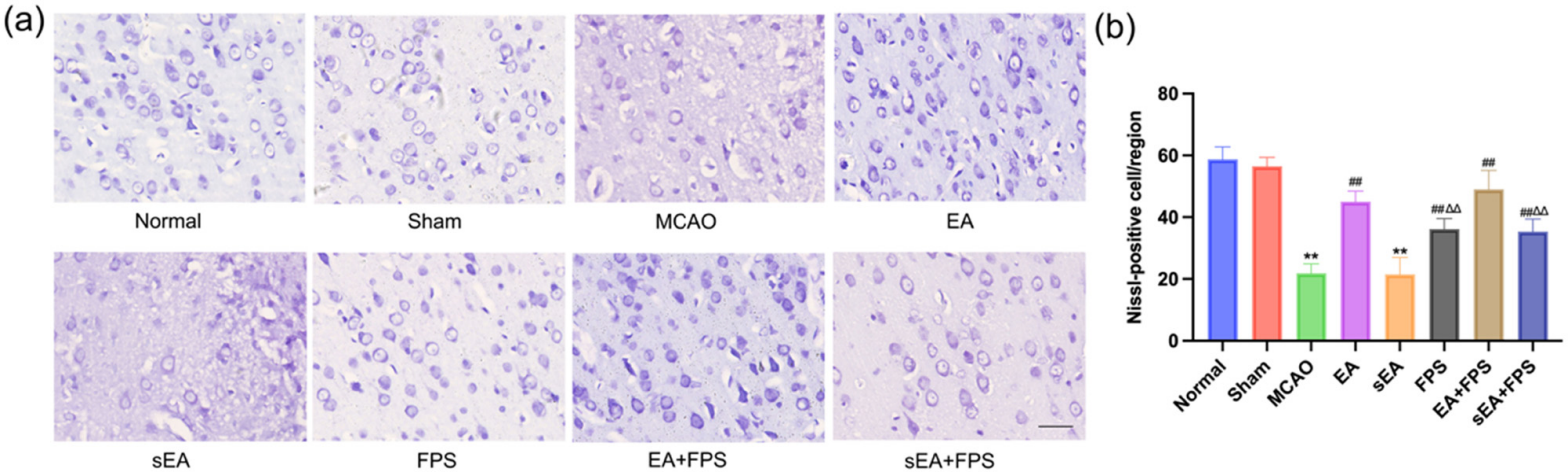
Nerve injury in the M1 region of the rat cortex in the eight groups on the 14th day after the treatment. (a) Representative micrographs of Nissl staining results. (b) Histogram of comparison of Nissl staining results. *n* = 6/group. Scale bar = 50 µm, magnification ×400. **p < 0.0001, MCAO vs Normal; **p < 0.0001, sEA vs Normal; ^##^ *p* < 0.0001, EA vs MCAO; ^##^ *p* < 0.0001, FPS vs MCAO; ^##^ *p* < 0.0001, EA + FPS vs MCAO; ^##^ *p* < 0.0001, sEA + FPS vs MCAO; ^∆∆^ *p* = 0.0001, FPS vs EA + FPS; ^∆∆^ *p* < 0.0001, sEA + FPS vs EA + FPS. ANOVA; mean ± SEM.

The morphology of neurons in the M1 region of the rat cortex was examined using Nissl staining, which stains neuronal nuclei blue. The number of neurons in each group was quantified and compared. Compared to the Normal group, both the MCAO and sEA groups exhibited a significant reduction in the number of neurons (*p* < 0.01). Conversely, the EA, FPS, EA + FPS, and sEA + FPS groups demonstrated a significant increase in the number of neurons compared to the MCAO group (*p* < 0.01). These findings indicate that treatment with EA and FPS-ZM1 resulted in a higher number of neurons in MCAO rats. Notably, the EA + FPS group exhibited a significantly higher number of neurons than both the FPS and sEA + FPS groups (*p* < 0.01) (Figure 2).

### 3.3 Results of immunofluorescence staining

**Figure 3:**
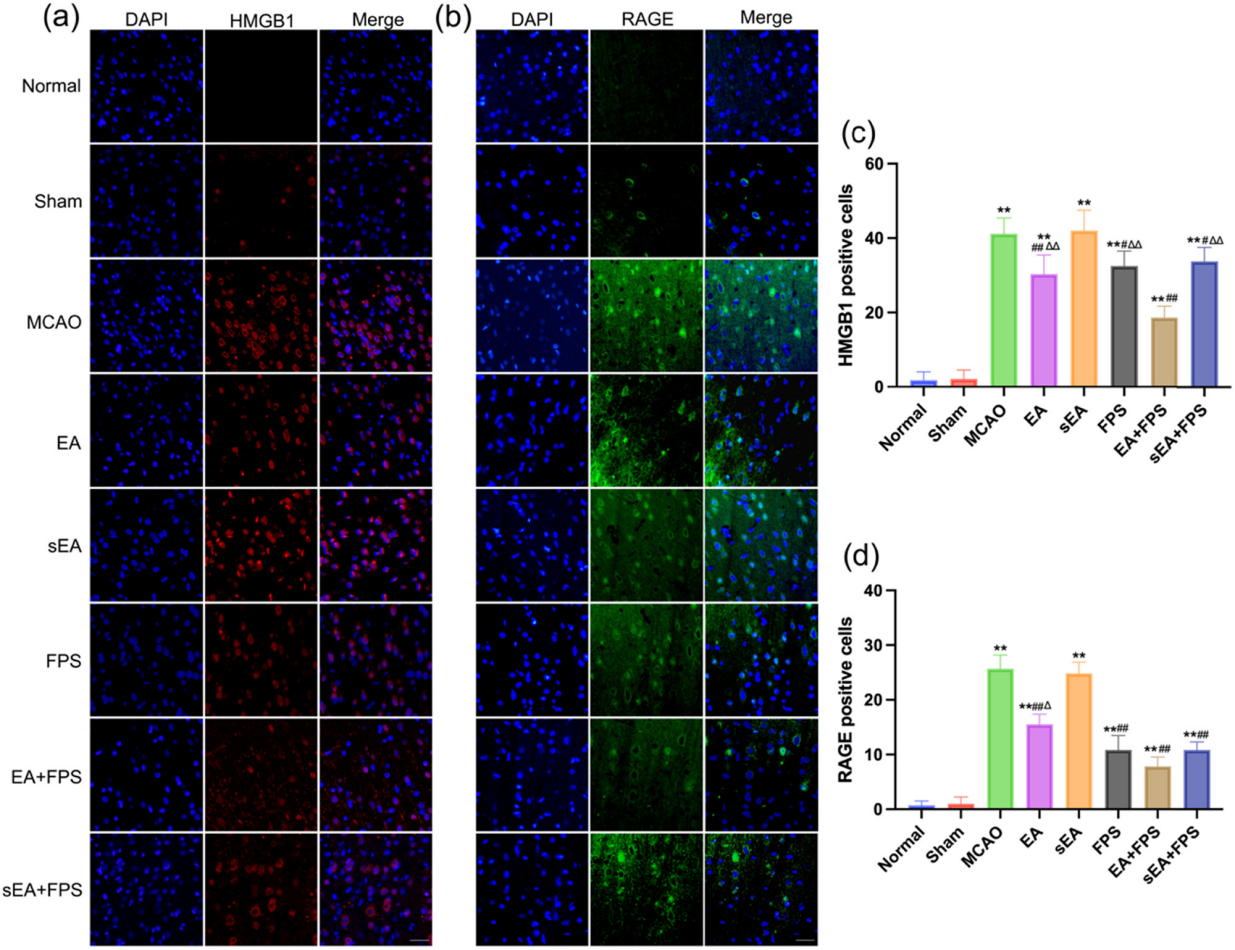
Immunofluorescence staining of HMGB1 and RAGE in the eight groups on the 14th day after the treatment. (a) Immunofluorescence staining of HMGB1 in the M1 region. (b) Immunofluorescence staining of RAGE in the M1 region. (c) An immunofluorescent analysis of HMGB1. *n* = 6/group. **p < 0.0001, MCAO vs Normal; **p < 0.0001, EA vs Normal; **p < 0.0001, sEA vs Normal; **p < 0.0001, FPS vs Normal; **p < 0.0001, EA + FPS vs Normal; **p < 0.0001, sEA + FPS vs Normal; ^##^ *p* < 0.0001, EA vs MCAO; ^##^ *p* < 0.0001, FPS vs MCAO; ^##^ *p* < 0.0001, EA + FPS vs MCAO; ^##^ *p* < 0.0001, sEA + FPS vs MCAO; ^∆∆^ *p* = 0.0007, EA vs EA + FPS; ^∆∆^ *p* < 0.0001, FPS vs EA + FPS; ^∆∆^ *p* < 0.0001, sEA + FPS vs EA + FPS. (d) An immunofluorescence analysis of RAGE. *n* = 6/group. Scale bar = 50 µm, magnification ×400. **p < 0.0001, MCAO vs Normal; **p < 0.0001, EA vs Normal; **p < 0.0001, sEA vs Normal; **p < 0.0001, FPS vs Normal; **p < 0.0001, EA + FPS vs Normal; **p < 0.0001, sEA + FPS vs Normal; ^##^ *p* < 0.0001, EA vs MCAO; ^##^ *p* < 0.0001, FPS vs MCAO; ^##^ *p* < 0.0001, EA + FPS vs MCAO; ^##^ *p* < 0.0001, sEA + FPS vs MCAO; ^∆∆^ *p* = 0.0064, EA vs EA + FPS. ANOVA; mean ± SEM.

The results of HMGB1 immunofluorescence staining revealed no significant difference between the Normal and Sham groups. However, there was a significant increase in the number of HMGB1-positive cells in the MCAO, EA, sEA, FPS, EA + FPS, and sEA + FPS groups compared to the Normal group (*p* < 0.01). In contrast, the EA, FPS, EA + FPS, and sEA + FPS groups exhibited a significant decrease in the number of HMGB1-positive cells compared to the MCAO group (*p* < 0.05), while no significant difference was observed between the sEA and MCAO groups. Notably, the number of HMGB1-positive cells increased in the EA, FPS, and sEA + FPS groups compared to the EA + FPS group (*p* < 0.01), with no significant difference between the EA, FPS, and sEA + FPS groups (Figure 3a and c).

Regarding RAGE immunofluorescence staining, no significant difference was found between the Normal and Sham groups. The number of RAGE-positive cells was significantly higher in the MCAO, EA, sEA, FPS, EA + FPS, and sEA + FPS groups than in the Normal group (*p* < 0.01). Conversely, the EA, FPS, EA + FPS, and sEA + FPS groups exhibited a significant decrease in the number of RAGE-positive cells compared to the MCAO group (*p* < 0.01), while no significant difference was observed between the sEA and MCAO groups. Furthermore, the EA group showed a significant increase in RAGE-positive cells compared to the EA + FPS group (*p* < 0.05), whereas no significant difference was observed between the FPS, EA + FPS, and sEA + FPS groups (Figure 3b and d).

### 3.4 Expression of HMGB1 and RAGE

**Figure 4:**
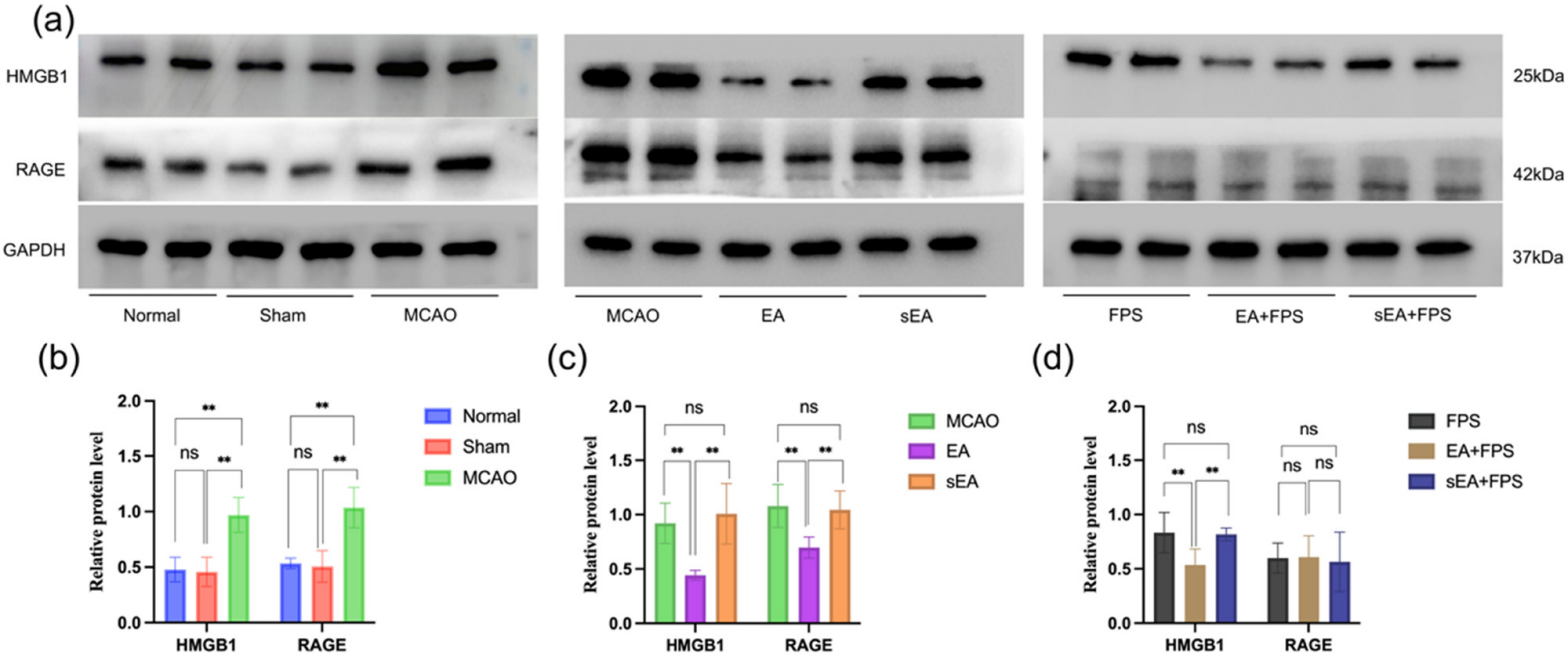
Expression of HMGB1 and RAGE proteins and statistical diagram of cerebral tissue in the M1 area of the right cortex in each group. (a) Protein expression of HMGB1 and RAGE in each group. (b) Statistical graph of HMGB1 and RAGE protein expression in the Normal, Sham, and MCAO groups. *n* = 6/group. HMGB1 statistical values: **p < 0.0001, MCAO vs Normal; **p < 0.0001, MCAO vs Sham. RAGE statistical values: **p < 0.0001, MCAO vs Normal; **p < 0.0001, MCAO vs Sham. (c) Statistical graph of HMGB1 and RAGE protein expression in the MCAO, EA, and sEA groups. *n* = 6/group. HMGB1 statistical values: **p = 0.0019, EA vs MCAO; **p = 0.0004, EA vs sEA. RAGE statistical values: **p = 0.0027, EA vs MCAO; **p = 0.0057, EA vs sEA. (d) Statistical graph of HMGB1 and RAGE protein expression in the FPS, EA + FPS, and sEA + FPS groups. *n* = 6/group. HMGB1 statistical values: **p = 0.0063, FPS vs EA + FPS; **p = 0.0092, EA + FPS vs sEA + FPS. ANOVA; mean ± SEM.

The western blot results revealed no significant difference in the relative expression of HMGB1 and RAGE between the Sham and Normal groups in the M1 region of the right cerebral cortex. However, a substantial increase in the relative expression of HMGB1 and RAGE was observed in the MCAO group compared to the Normal group (*p* < 0.01) (Figure 4a and b). Notably, the EA group exhibited significantly lower levels of HMGB1 and RAGE expression than the MCAO and sEA groups (*p* < 0.01). However, there were no significant differences in HMGB1 and RAGE expression levels between the sEA and MCAO groups (Figure 4a and c). Additionally, the EA + FPS group showed significantly lower HMGB1 expression in the M1 region of the right cortex than the FPS and sEA + FPS groups (*p* < 0.01). However, no noticeable differences in RAGE expression levels were observed between the FPS group and the EA + FPS and sEA + FPS groups (Figure 4a and d).

## 4 Discussion

Acupuncture plays a significant role in various stages of the stroke cascade, beginning with the cellular damage caused by hypoxia and glucose deficiency. It affects multiple interconnected molecular pathways, including excitotoxicity, acidosis, ion imbalance, hyperglycemia, and inflammation [20]. Numerous studies have investigated the mechanisms underlying the neuroprotective effects of EA therapy. These studies have demonstrated that EA improves neurological outcomes [21], reduces neural inflammation and hypoxia [22,23], and facilitates angiogenesis and neurogenesis [24–27]. Based on these findings, we selected GV20 and ST36 as the acupuncture points for treating the MCAO rats.

This study revealed a decrease in neurological function scores and a significant reduction in the number of neuronal cells in the MCAO group compared to the control group. Immunofluorescence staining and western blot demonstrated significantly higher levels of HMGB1 and RAGE proteins in the MCAO group. Notably, HMGB1 protein function is determined by its redox status, with the reduced form acting as a chemoattractant and the oxidized form triggering the release of inflammatory cytokines [28]. Following cerebral ischemic injury, extracellular release of HMGB1 exacerbates the inflammatory response through autocrine or paracrine mechanisms [29]. Previous research has indicated that HMGB1 promotes platelet aggregation and accelerates thrombosis upon secretion [30]. RAGE, a receptor for HMGB1, was initially isolated from bovine lung and consists of three extracellular immunoglobulin structural domains (V, C1, and C2), with the V region serving as the primary ligand-binding domain. While RAGE expression is low in healthy human tissues, its levels are increased in various inflammatory and vascular diseases [31]. Thus, targeting the HMGB1/RAGE pathway holds promise as a therapeutic approach for cerebral ischemia.

Acupuncture treatment at the GV20 and ST36 acupoints demonstrated a neuroprotective effect in MCAO rats [27,32]. Our study revealed a significant improvement in neurological function scores and Nissl staining results in the EA group compared to the MCAO and sEA groups, with no significant difference observed between the MCAO and sEA groups. Animal experiments have shown that EA promotes neuronal regeneration, enhances motor cortical excitability, stimulates cerebral angiogenesis, mitigates motor dysfunction, reduces brain edema, and preserves the integrity of the blood–brain barrier [33,34]. Previous studies have also highlighted the protective effects of EA following ischemic stroke, such as modulating microglial polarization, suppressing inflammation, improving motor function through the PI3K/AKT signaling pathway, and regulating the mTOR signaling pathway via motor cortex activation [35–38]. Additionally, our results demonstrated a significant reduction in the expression of HMGB1/RAGE in the EA group compared to both the MCAO and sEA groups, aligning with previous reports on the effects of EA. These reports have shown that EA can ameliorate cognitive impairment in mouse models of Alzheimer’s disease by inhibiting fibrin/Aβ deposition and deactivating the HMGB1/RAGE signaling pathways, as well as alleviate neuroinflammation by downregulating hippocampal HMGB1/RAGE signaling [39,40]. Therefore, our findings suggest that acupoint EA may mitigate neurological impairment resulting from cerebral ischemia through modulation of the HMGB1/RAGE pathway.

The highly selective RAGE inhibitor, FPS-ZM1, has been shown to alleviate neuroinflammation in focal cerebral ischemia rats by suppressing astrocyte activation and microglial cell proliferation, and reducing proinflammatory cytokine levels [5,13]. Our study using neurological function scores and Nissl staining confirmed a significant reduction in neurological deficits and neuronal cell death in rats treated with FPS-ZM1. Some studies have shown that both EA and FPS-ZM1 can reduce the infarct size in cerebral ischemic rats [9,41], and the combination of EA and other therapeutic means can reduce the infarct size more significantly [21,42].

Moreover, the combination of EA and FPS-ZM1 demonstrated synergistic effect in reducing ischemic symptoms compared to individual treatments alone, indicating that EA exerts neuroprotective effects through mechanisms beyond the inhibition of the HMGB1/RAGE pathway. EA has multi-level, multi-pathway, and multi-factor advantages [12]. The synergistic effect shown by EA and FPS-ZM1 compared with each single intervention group may be related to the fact that EA could alter the cellular response or sensitivity to the drug by affecting the absorption, distribution, and metabolism of FPS-ZM1 *in vivo*, and it may be related to the fact that EA can alter cellular response or sensitivity to drugs by affecting the specific receptor on the cells of the target organ and signaling pathways other than HMGB1/RAGE to alter cellular response or sensitivity to drugs. This is also a worthy next step to explore.

Our findings emphasize the crucial involvement of HMGB1 and RAGE in mediating cerebral ischemia-induced damage in the M1 region of the cerebral cortex. EA was found to mitigate neurological damage by inhibiting the HMGB1/RAGE pathway and its neuroprotective effects through other pathways. These novel insights into the pathogenesis of cerebral ischemia highlight the potential of targeting the HMGB1/RAGE pathway with EA as a promising therapeutic strategy for this debilitating condition.

## 5 Conclusions

The HMGB1/RAGE pathway is activated after cerebral ischemia. EA and FPS-ZM1 can improve neurological deficits and reduce neuronal injury. The combination of EA and FPS-ZM1 demonstrated synergistic effect in reducing ischemic symptoms. EA can exert neuroprotective effects by inhibiting the activation of HMGB1/RAGE. These findings provide valuable insights into the use of EA as a clinical approach for the treatment of ischemic stroke.

## Supplementary Material

Supplementary Figure

## References

[1] Feske SK. Ischemic stroke. Am J Med. 2021;134:1457–64. 10.1016/j.amjmed.2021.07.027 34454905

[2] Liang Y, Song P, Chen W, Xie X, Luo R, Su J, et al. Inhibition of caspase-1 ameliorates ischemia-associated blood–brain barrier dysfunction and integrity by suppressing pyroptosis activation. Front Cell Neurosci. 2020;14:540669. 10.3389/fncel.2020.540669. PMC7874210 33584203

[3] Fan H, Tang HB, Chen Z, Wang HQ, Zhang L, Jiang Y, et al. Inhibiting HMGB1-RAGE axis prevents pro-inflammatory macrophages/microglia polarization and affords neuroprotection after spinal cord injury. J Neuroinflammation. 2020;17:295. 10.1186/s12974-020-01973-4 PMC7547440 33036632

[4] Brett J, Schmidt AM, Yan SD, Zou YS, Weidman E, Pinsky D, et al. Survey of the distribution of a newly characterized receptor for advanced glycation end products in tissues. Am J Pathol. 1993;143:1699–712. PMC1887265 8256857

[5] Mi L, Zhang Y, Xu Y, Zheng X, Zhang X, Wang Z, et al. HMGB1/RAGE pro-inflammatory axis promotes vascular endothelial cell apoptosis in limb ischemia/reperfusion injury. Biomed Pharmacother. 2019;116:109005. 10.1016/j.biopha.2019.109005 31136947

[6] Shen C, Ma Y, Zeng Z, Yin Q, Hong Y, Hou X, et al. RAGE-specific inhibitor FPS-ZM1 attenuates AGEs-induced neuroinflammation and oxidative stress in rat primary microglia. Neurochem Res. 2017;42:2902–11. 10.1007/s11064-017-2321-x 28664403

[7] Deane R, Singh I, Sagare AP, Bell RD, Ross NT, LaRue B, et al. A multimodal RAGE-specific inhibitor reduces amyloid β-mediated brain disorder in a mouse model of Alzheimer disease. J Clin Invest. 2012;122:1377–92. 10.1172/JCI58642 PMC3314449 22406537

[8] Gasparotto J, Ribeiro CT, Bortolin RC, Somensi N, Rabelo TK, Kunzler A, et al. Targeted inhibition of RAGE in substantia nigra of rats blocks 6-OHDA-induced dopaminergic denervation. Sci Rep. 2017;7:8795. 10.1038/s41598-017-09257-3 PMC5562811 28821831

[9] Shen L, Zhang T, Yang Y, Lu D, Xu A, Li K. FPS-ZM1 alleviates neuroinflammation in focal cerebral ischemia rats via blocking ligand/RAGE/DIAPH1 pathway. ACS Chem Neurosci. 2021;12:63–78. 10.1021/acschemneuro.0c00530 33300334

[10] Singh H, Agrawal DK. Therapeutic potential of targeting the receptor for advanced glycation end products (RAGE) by small molecule inhibitors. Drug Dev Res. 2022;83:1257–69. 10.1002/ddr.21971 PMC9474610 35781678

[11] Wen J, Chen X, Yang Y, Liu J, Li E, Liu J, et al. Acupuncture medical therapy and its underlying mechanisms: a systematic review. Am J Chin Med. 2021;49:1–23. 10.1142/S0192415X21500014 33371816

[12] Chavez LM, Huang SS, MacDonald I, Lin JG, Lee YC, Chen YH. Mechanisms of acupuncture therapy in ischemic stroke rehabilitation: a literature review of basic studies. Int J Mol Sci. 2017;18:2270. 10.3390/ijms18112270 PMC5713240 29143805

[13] Xu XY, Fang Q, Huang W, Li BC, Zhou XH, Zhou ZY, et al. Effect of electroacupuncture on neurological deficit and activity of clock and Bmal1 in cerebral ischemic rats. Curr Med Sci. 2020;40:1128–36. 10.1007/s11596-020-2295-9 33428141

[14] Yang Y, Deng P, Si Y, Xu H, Zhang J, Sun H. Acupuncture at GV20 and ST36 improves the recovery of behavioral activity in rats subjected to cerebral ischemia/reperfusion injury. Front Behav Neurosci. 2022;16:909512. 10.3389/fnbeh.2022.909512 PMC9239252 35775011

[15] Xu H, Zhang YM, Sun H, Chen SH, Si YK. Electroacupuncture at GV20 and ST36 exerts neuroprotective effects via the EPO-mediated JAK2/STAT3 pathway in cerebral ischemic rats. Evid Based Complement Altern Med. 2017;2017:6027421. 10.1155/2017/6027421 PMC5564076 28848617

[16] Benedetti B, Dannehl D, Janssen JM, Corcelli C, Couillard-Després S, Engelhardt M. Structural and functional maturation of rat primary motor cortex layer V neurons. Int J Mol Sci. 2020;21:6101. 10.3390/ijms21176101 PMC7503395 32847128

[17] Li Y, Wang D, Zhang H, Wang Y, Wu P, Zhang H, et al. Changes of brain connectivity in the primary motor cortex after subcortical stroke: a multimodal magnetic resonance imaging study. Medicine (Baltimore). 2016;95:e2579. 10.1097/MD.0000000000002579 PMC4753872 26871777

[18] Zhang YM, Xu H, Chen SH, Sun H. Electroacupuncture regulates endoplasmic reticulum stress and ameliorates neuronal injury in rats with acute ischemic stroke. Evid Based Complement Altern Med. 2021;2021:9912325. 10.1155/2021/9912325 PMC8382524 34434247

[19] Chen ZX, Li Y, Zhang XG, Chen S, Yang WT, Zheng XW, et al. Sham electroacupuncture methods in randomized controlled trials. Sci Rep. 2017;7:40837. 10.1038/srep40837 PMC5247761 28106094

[20] Wang JX, Ma LX, Mu JD, Sun TY, Qian X, Yu WY, et al. Anti-spastic effect induced by waggle needling correlates with KCC2-GABAA pathway in post-stroke spasticity rats. Neurosci Lett. 2021;750:135810. 10.1016/j.neulet.2021.135810 33705929

[21] Deng P, Wang L, Zhang Q, Chen S, Zhang Y, Xu H, et al. Therapeutic potential of a combination of electroacupuncture and human iPSC-derived small extracellular vesicles for ischemic stroke. Cells. 2022;11:820. 10.3390/cells11050820 PMC8909871 35269441

[22] Long M, Wang Z, Zheng D, Chen J, Tao W, Wang L, et al. Electroacupuncture pretreatment elicits neuroprotection against cerebral ischemia–reperfusion injury in rats associated with transient receptor potential vanilloid 1-mediated anti-oxidant stress and anti-inflammation. Inflammation. 2019;42:1777–87. 10.1007/s10753-019-01040-y 31190106

[23] Xu H, Qin W, Hu X, Mu S, Zhu J, Lu W, et al. Lentivirus-mediated overexpression of OTULIN ameliorates microglia activation and neuroinflammation by depressing the activation of the NF-κB signaling pathway in cerebral ischemia/reperfusion rats. J Neuroinflammation. 2018;15:83. 10.1186/s12974-018-1117-5 PMC5856386 29544517

[24] Shi L, Cao HM, Li Y, Xu SX, Zhang Y, Zhang Y, et al. Electroacupuncture improves neurovascular unit reconstruction by promoting collateral circulation and angiogenesis. Neural Regen Res. 2017;12:2000–6. 10.4103/1673-5374.221156 PMC5784347 29323038

[25] Li Z, Meng X, Ren M, Shao M. Combination of scalp acupuncture with exercise therapy effectively counteracts ischemic brain injury in rats. J Stroke Cerebrovasc Dis. 2020;29:105286. 10.1016/j.jstrokecerebrovasdis.2020.105286 33066914

[26] Li J, Chen L, Li D, Lu M, Huang X, Han X, et al. Electroacupuncture promotes the survival of the grafted human MGE neural progenitors in rats with cerebral ischemia by promoting angiogenesis and inhibiting inflammation. Neural Plast. 2021;2021:4894881. 10.1155/2021/4894881 PMC8516583 34659396

[27] Chen S, Wang H, Xu H, Zhang Y, Sun H. Electroacupuncture promotes axonal regrowth by attenuating the myelin-associated inhibitors-induced RhoA/ROCK pathway in cerebral ischemia/reperfusion rats. Brain Res. 2020;1748:147075. 10.1016/j.brainres.2020.147075 32853644

[28] Vogel S, Bodenstein R, Chen Q, Feil S, Feil R, Rheinlaender J, et al. Platelet-derived HMGB1 is a critical mediator of thrombosis. J Clin Invest. 2015;125:4638–54. 10.1172/JCI81660 PMC4665785 26551681

[29] Li C, Sun T, Jiang C. Recent advances in nanomedicines for the treatment of ischemic stroke. Acta Pharm Sin B. 2021;11:1767–88. 10.1016/j.apsb.2020.11.019 PMC8343119 34386320

[30] Stark K, Philippi V, Stockhausen S, Busse J, Antonelli A, Miller M, et al. Disulfide HMGB1 derived from platelets coordinates venous thrombosis in mice. Blood. 2016;128:2435–49. 10.1182/blood-2016-04-710632 PMC5147023 27574188

[31] Hudson BI, Lippman ME. Targeting RAGE signaling in inflammatory disease. Annu Rev Med. 2018;69:349–64. 10.1146/annurev-med-041316-085215 29106804

[32] Wang H, Chen S, Zhang Y, Xu H, Sun H. Electroacupuncture ameliorates neuronal injury by Pink1/Parkin-mediated mitophagy clearance in cerebral ischemia–reperfusion. Nitric Oxide. 2019;91:23–34. 10.1016/j.niox.2019.07.004 31323277

[33] Yu BH, Xing Y, Zhang F. The therapeutic effect of electroacupuncture therapy for ischemic stroke. Evid Based Complement Altern Med. 2020;2020:6415083. 10.1155/2020/6415083 PMC7718040 33293991

[34] Chen B, Lin WQ, Li ZF, Zhong XY, Wang J, You XF, et al. Electroacupuncture attenuates ischemic brain injury and cellular apoptosis via mitochondrial translocation of cofilin. Chin J Integr Med. 2021;27:705–12. 10.1007/s11655-021-3335-4 33709239

[35] Chen S, Wang L, Yuan Y, Wen Y, Shu S. Electroacupuncture regulates microglia polarization via lncRNA-mediated hippo pathway after ischemic stroke. Biotechnol Genet Eng Rev. 2023;39:1–17. 10.1080/02648725.2023.2177046 36760060

[36] Wang Y, Chen Y, Meng L, Wu B, Ouyang L, Peng R, et al. Electro-acupuncture treatment inhibits the inflammatory response by regulating γδ T and Treg cells in ischemic stroke. Exp Neurol. 2023;362:114324. 10.1016/j.expneurol.2023.114324 36669751

[37] Zhang XQ, Wang YH, Sun L, Dong BQ, Sui YJ, Dong JZ, et al. Electroacupuncture promotes motor function recovery in MCAO/R rats by activating astrocyte-related PI3K/AKT pathway. J Acupunct Meridian Stud. 2022;15:322–32. 10.51507/j.jams.2022.15.5.322 36521830

[38] Zhang Y, Yin YL, Jin ZY, Hu QP, Wu XG. Electroacupuncture activates neuroplasticity in the motor cortex and corticospinal tract via the mTOR pathway in a rat p-MCAO model. Biomed Res Int. 2022;2022:3470685. 10.1155/2022/3470685 PMC9683956 36440366

[39] Wang Y, Wang Q, Luo D, Zhao P, Zhong SS, Dai B, et al. Electroacupuncture improves blood–brain barrier and hippocampal neuroinflammation in SAMP8 mice by inhibiting HMGB1/TLR4 and RAGE/NADPH signaling pathways. Chin J Integr Med. 2023;29:448–58. 10.1007/s11655-023-3592-5 36609953

[40] Xin Y, Wang J, Chu T, Zhou Y, Liu C, Xu A. Electroacupuncture alleviates neuroinflammation by inhibiting the HMGB1 signaling pathway in rats with sepsis-associated encephalopathy. Brain Sci. 2022;12:1732. 10.3390/brainsci12121732 PMC9776077 36552192

[41] Sha R, Zhang B, Han X, Peng J, Zheng C, Zhang F, et al. Electroacupuncture alleviates ischemic brain injury by inhibiting the miR-223/NLRP3 pathway. Med Sci Monit. 2019;25:4723–33. 10.12659/MSM.917213 PMC6607941 31237865

[42] Yu Q, Li X, Li Y, Fu J, Xiao Z. Effects of combined electroacupuncture and exercise training on motor function and microtubule-associated protein 2 expression in the middle and late stages of cerebral infarction in rats. Acupunct Med. 2020;38(3):175–80. 10.1177/0964528419882937 31996007

