# Supplementary figures and images for: Electroacupuncture inhibits the expression of HMGB1/RAGE and alleviates injury to the primary motor cortex in rats with cerebral ischemia
Source: Transl Neurosci. 2023 Oct 9;14(1):20220316. doi: 10.1515/tnsci-2022-0316 (PMC10566473; doi:10.1515/tnsci-2022-0316)

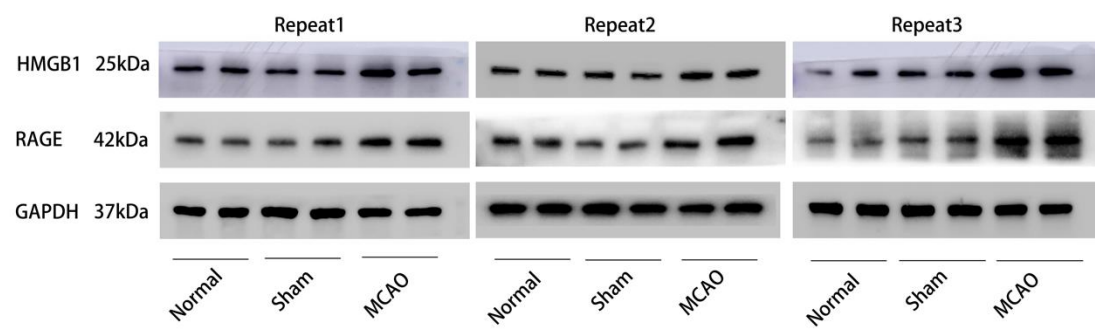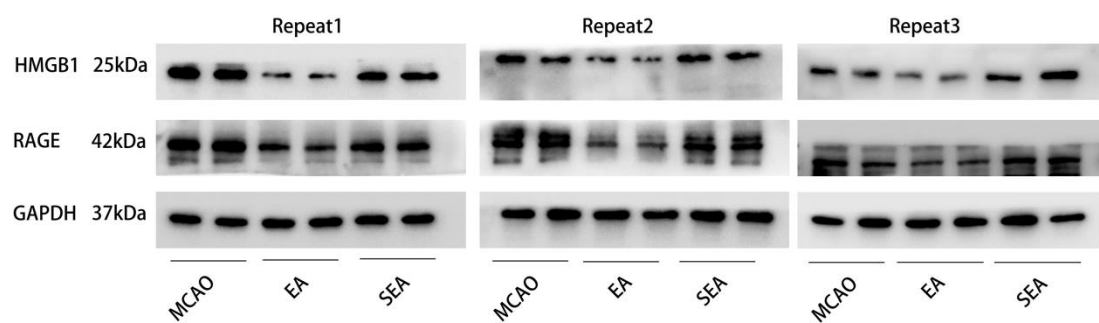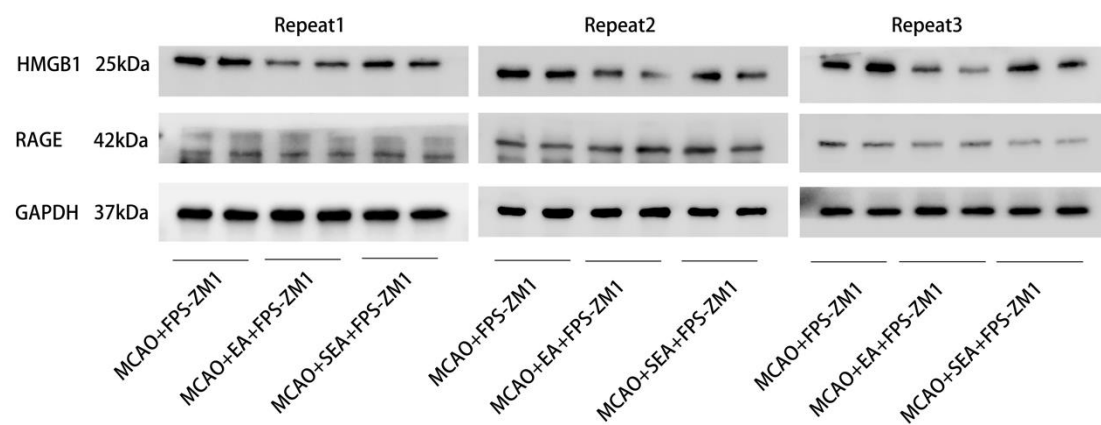

Supplement: Supplementary Figure [file tnsci-2022-0316-sm.pdf]
